# Biocompatibility of Intracanal Medications Based on Calcium Hydroxide

**DOI:** 10.5402/2012/904963

**Published:** 2012-12-18

**Authors:** Carolina Andolfatto, Guilherme Ferreira da Silva, Ana Livia Gomes Cornélio, Juliane Maria Guerreiro-Tanomaru, Mario Tanomaru-Filho, Gisele Faria, Idomeo Bonetti-Filho, Paulo Sérgio Cerri

**Affiliations:** ^1^Department of Restorative Dentistry, Araraquara Dental School, Universidade Estadual Paulista (UNESP), 14801-903 Araraquara, SP, Brazil; ^2^Laboratory of Histology and Embryology, Department of Morphology, Araraquara Dental School, Universidade Estadual Paulista (UNESP), 14801-903 Araraquara, SP, Brazil

## Abstract

*Objective*. The aim of this study was to evaluate the rat subcutaneous tissue reaction to calcium hydroxide-based intracanal medicaments, UltraCal XS (calcium hydroxide, barium sulphate, aqueous matrix), Hydropast (calcium hydroxide, barium sulphate, and propyleneglycol), and Calen (Calcium hydroxide, zinc oxide, colophony, and polyethyleneglycol), used as a control. *Methods*. Forty-eight rats (*Rattus Norvegicus Holtzman*) were distributed in three groups: Calen, UltraCal XS, and Hydropast. Polyethylene tubes filled with one of the medicaments were implanted in the dorsal subcutaneous. After 7 and 30 days, the implants were removed and the specimens were fixed and embedded in paraffin. Morphological and quantitative analyses were carried out in the HE-stained sections. The numerical density of inflammatory cells in the capsule was evaluated and statistical analyses were performed (*P* ≤ 0.05). *Results*. At 7 days, all materials induced an inflammatory reaction in the subcutaneous tissue adjacent to the implants. In all groups, a significant reduction in the number of inflammatory cells and giant cells was verified in the period of 30 days. *Conclusion*. These results indicate that the calcium hydroxide-based medicaments evaluated present biocompatibility similar to Calen.

## 1. Introduction

The success of endodontic treatment of teeth with periapical lesion depends on the reduction or elimination of the intraradicular infection [1, 2]. The root canal mechanical preparation is not enough to eliminate this infection because many microorganisms are not only in the main root canal, but also disseminated throughout the root canal system. Therefore, the use of an intracanal dressing to eliminate the microorganisms is indicated [3–6].

Antimicrobial activity and biocompatibility are characteristics that an ideal intracanal dressing has to show [7]. Calcium hydroxide [Ca(OH)_2_] has been widely used for its biological and antimicrobial activity [4, 8], ability to dissolve organic tissue [9], and capacity to inactivate bacterial endotoxin [10, 11]. Despite these properties, the Ca(OH)_2_ has no satisfactory physical properties such as radiopacity to visualize on dental radiographs and flow capacity to facilitate its insertion in the root canal [12, 13]. For this reason, it needs the incorporation of a radiopacifying agent and a vehicle to improve these characteristics [8, 14]. 

Although the Ca(OH)_2_ shows an excellent biocompatibility, the addition of other substances can affect its biological properties [8, 12]. In the last years, it has been marketed the calcium hydroxide-based medicaments such as UltraCal XS (Ultradent Products, Inc.) and Hydropast (Biodinâmica Química e Farmacêutica Ltda., Brazil). UltraCal XS is basically composed by 35% of calcium hydroxide, a radiopacifier, and a vehicle which does have not the proportions related by the manufacturer. Studies have demonstrated that UltraCal XS has a high pH value [15] and an effective antimicrobial activity against common endodontic bacteria of teeth with pulp necrosis [16]. On the other hand, Brazilian paste Hydropast, composed by 38% of calcium hydroxide, barium oxide, as a radiopacifying agent, and propilenoglycol as vehicle, is a recent material, and, therefore, until now, there are no studies of its biological properties. 

Considering the recommendation of International Organization for Standardization [17], it is necessary *in vitro* and/or *in vivo* studies for evaluation of the biocompatibility of these new materials. Thus, the purpose of this study was to evaluate the tissue reaction of these calcium hydroxide-based medicaments in rat subcutaneous.

## 2. Materials and Methods

### 2.1. Animals and Experimental Proceedings

This study was performed in accordance with the principles of animal care on animal experiments. The research protocol was authorized by the Ethical Committee for Animal Research of the São Paulo State University, Brazil (Dental School, UNESP, Araraquara).

Forty-eight male Holtzman rats (*Rattus norvegicus albinus*), weighing 250 ± 10 g, were kept in individual stainless steel cages under 12 : 12 light-dark cycle at controlled temperature (23 ± 2°C) and humidity (55 + 10%), with food and water provided *ad libitum*. The animals were randomly distributed into three groups (*n* = 16) on the basis of the intracanal medicaments analysed, Calen group, Calen paste (S. S. White Artigos Dentários Ltda., Rio de Janeiro, RJ, Brazil), used as control group; UltraCal group, UltraCal XS paste (Ultradent Products, Inc., South Jordan, UT, USA), and Hydropast group, Hydropast (Biodinâmica Química e Farmacêutica Ltda., Ibipora, PR, Brazil). The composition of these materials is described at Table 1. 

The polyethylene tubes (Embramed Ind. Com. Ltda., São Paulo, SP, Brazil) with 10.0 mm length and 1.5 mm diameter previously sterilized with ethylene oxide were filled with Calen, UltraCal XS paste, or Hydropast intracanal medicaments. 

The animals were anaesthetized with an intraperitoneal injection containing 80 mg/Kg of body weight of ketamine (União Química Farmacêutica Nacional S/A-Brazil) and 4 mg/Kg of body weight of xylazine (Virbac do Brasil Indústria e Comércio Ltda., Brazil). After shaved and disinfection with 5% iodine solution, a 20 mm-long incision in a head-to-tail orientation was made using a scalpel (no. 15, Fibra Cirúrgica, Joinvile, SC, Brazil) in the dorsal skin. Subsequently, the polyethylene tube containing Calen paste, UltraCal XS, or Hydropast was immediately implanted into the dorsal subcutaneous connective tissue. After implantation, the skin was closed with 4.0 silk suture (Vicryl; Johnson & Johnson: Ethicon Inc., New Brunswick, NJ, USA). One polyethylene tube filled with an intracanal paste was implanted in each animal and left for periods of 7 and 30 days.

After experimental periods, the animals were killed by overdose of anesthetic solution, and the tubes were removed with surrounding connective tissue and prepared for paraffin embedding.

### 2.2. Histological Procedures and Analysis

The specimens containing the implanted polyethylene tubes were fixed in 4% formaldehyde (prepared from paraformaldehyde) buffered at pH 7.2 with 0.1 M sodium phosphate at room temperature for 48 hours. Subsequently, the specimens were dehydrated and embedded in paraffin. Serial 6 *μ*m-thick sections were made parallel to the tube long axis and stained with hematoxylin and eosin (H&E) for morphological and morphometric analyses. The morphological analysis of the capsule in contact with the material on the opening of the tube was performed considering the following parameters: presence of inflammatory process, main cells (inflammatory cells or fibroblasts) present in the capsule, presence of multinucleated giant cells, and presence of collagen fibers. 

 The numerical density of inflammatory mononucleated cells and multinucleated giant cells was undertaken using a light microscope (BX51, Olympus, Tokyo, Japan) and an image analysis system (Image Pro-Express 6.0, Olympus). Three H&E-stained sections per animal were selected at intervals of at least 100 *μ*m; in each section, a standardized field of 0.09 mm^2^ of the connective tissue adjacent to the opening of the tube implanted was analyzed, totaling 0.27 mm^2^ per animal. In each area, the total number of inflammatory cells was counted using the image analysis system at ×40 magnification; in each animal, the total number of inflammatory cells was divided by total area, and, then, the number of inflammatory cells/mm^2^ was obtained. The differences between the groups were statistically analyzed using SigmaStat 2.0 software (Jandel Scientific, Sausalito, CA, USA); the data were submitted to ANOVA and Tukey test. The significance level accepted was *P* ≤ 0.05.

## 3. Results

### 3.1. Morphological and Quantitative Analyses

**Figure 1:**
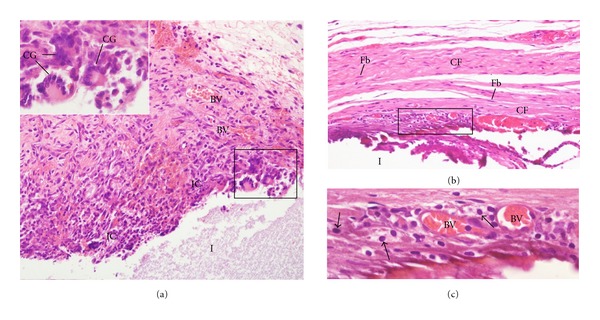
Light micrographs of sections showing portions of capsules adjacent to the opening of the tubes (I) filled with Calen paste after 7 (a) and 30 days (b and c) of implantation in the subcutaneous. In (a), numerous inflammatory cells (ICs) are observed in the inner portion of the capsule adjacent to the tube opening (I). The inset of the outlined area shows multinucleated giant cells (GCs) in close juxtaposition to the material implanted. BV, blood vessels. ×110; inset: ×250 (b) shows the capsule exhibits several fibroblasts (Fb) dispersed among the collagen fiber bundles (CF) ×130. In (c), outlined area in (b), shows some inflammatory cells (arrows), mainly lymphocytes, situated adjacent to the blood vessels (BV) ×250.

**Figure 2:**
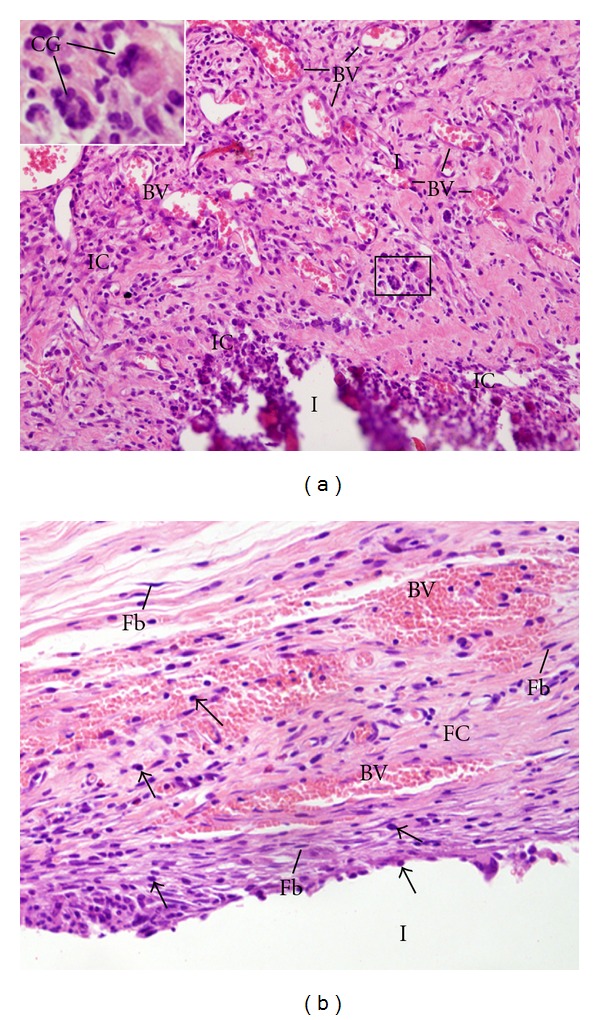
Light micrographs of sections showing portions of capsules adjacent to the opening of the tubes (I) filled with UltraCal XS paste after 7 (a) and 30 days (b) of implantation in the subcutaneous. In (a), The capsule exhibits several inflammatory cells (IC) and blood vessels (BV). Note that dense masses of inflammatory cells (ICs) are observed in the inner portion of the capsule, adjacent to the tube opening (I). The inset, outlined area, shows multinucleated giant cells (GCs). ×120; inset: ×230. (b) The capsule contains several fibroblasts (Fbs) and numerous blood vessels (BV). Inflammatory cells (arrows), lymphocytes and macrophages are situated mainly in the inner portion of the capsule and next to the blood vessels ×150.

**Figure 3:**
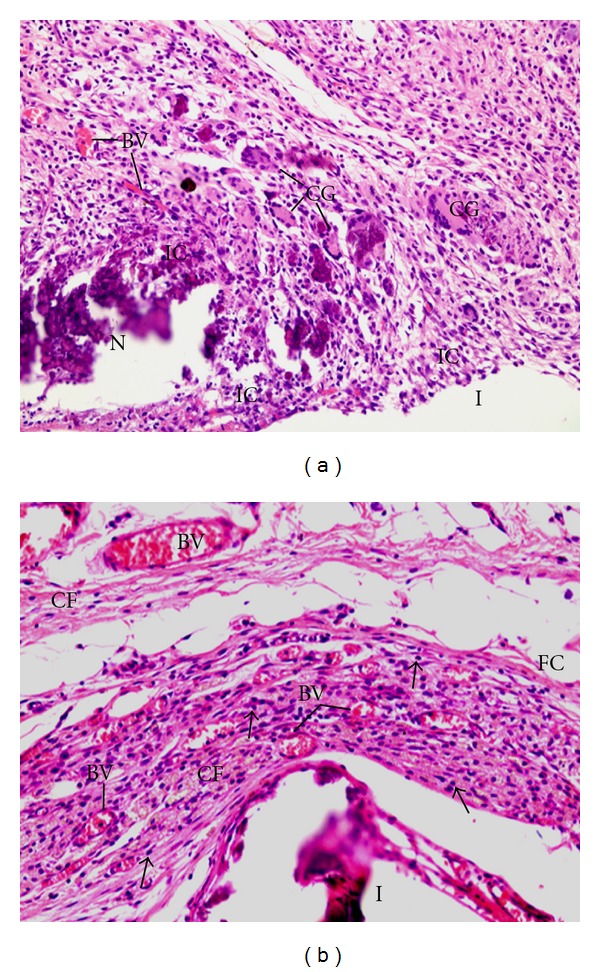
Light micrographs of sections showing portions of capsules adjacent to the opening of the tubes (I) filled with Hydropast after 7 (a) and 30 days (b) of implantation in the subcutaneous. In (a), numerous inflammatory cells (ICs) and multinucleated giant cells (GCs) are present by thorough capsule. BVs, blood vessels ×120. (b) The capsule contains predominantly inflammatory cells (arrows); scarce collagen fibers (FC) distributed irregularly are observed in the capsule. BVs, blood vessels ×120.

 After 7 days of implantation, the capsule adjacent to the opening of the tubes filled with Calen, UltraCal XS, and Hydropast paste showed numerous inflammatory cells, mainly lymphocytes and macrophages. Usually, the inflammatory infiltration was evident in the innermost portion of the capsule, that is, in close juxtaposition to the materials (Figures 1(a), 2(a) and 3(a)). According to Table 2, no significant difference was verified in the numerical density of inflammatory cells among the groups. Multinucleated giant cells were also observed in the capsules formed in all groups (Figures 1(a), 2(a), and 3(a)). However, in the Hydropast group the number of multinucleated giant cells was significantly higher in comparison to other groups; on the other hand, the capsule of the Calen group exhibited the lower number of multinucleated giant cells (Table 2).

A significant decrease in the number of inflammatory cells and multinucleated giant cells was verified from 7 to 30 days, in all groups (Figures 1(b), 2(b), and 3(b); Table 2). In the period of 30 days, the capsule adjacent to Calen paste was formed by a dense connective tissue exhibiting typical bundles of collagen fibers between fibroblasts; lymphocytes and plasma cells were mainly present next to blood vessels (Figure 1(b)). In the UltraCal group, the capsule contained several fibroblasts among the inflammatory cells; usually, bundles of collagen fibers were only present in the outermost portion of the capsule (Figure 2(b)). The connective tissue of the capsule of the Hydropast exhibited several cells and scarce collagen fibers (Figure 3(b)). Although no significant difference was found in the number of inflammatory cells between the groups, the mean of the numerical density of inflammatory cells in the Hydropast group was around 1,400 cells/mm^2^, whereas in the other groups was around 965 cells/mm^2^. Moreover, in the Calen group the number of multinucleated giant cells was significantly lower in comparison to UltraCal and Hydropast groups (Table 2). 

## 4. Discussion

Implantation in the subcutaneous connective tissues of experimental animals has been extensively used to evaluate the biocompatibility of endodontic materials [18, 19]. Our findings indicate that UltraCal XS and Hydropast exhibit biological behavior similar to Calen (control group). At 7 days, an intense inflammatory reaction and foci of coagulative necrosis were seen in the adjacent capsule to the implanted materials. After 30 days, significant reduction in the inflammatory process was verified in all analyzed groups; usually, the capsule formed juxtaposed to the Calen paste exhibited inflammatory cells among typical bundles of collagen fibers and fibroblasts. The inflammatory reaction observed in the period of the 7 days may be attributed to the superficial necrosis promoted by calcium hydroxide-based materials [20]. Calcium hydroxide has an alkaline pH [21] and, when in contact with the connective tissue, induces the formation of a coagulative necrosis zone [22]. Coagulative necrosis refers to a spectrum of morphological changes in living tissue resulting from the action of enzymes on lethally injured cells. The mass of necrotic cells is characterized by preservation of the basic outline of the coagulated cells for a span of at least some days. As necrotic cells are unable to maintain membrane integrity, their contents leak out and elicit an inflammatory response that removes the cellular debris by phagocytosis, followed by healing [20].

The vehicles mixed with calcium hydroxide powder play an important role in the ionic dissociation process and so in the disinfection of the root canal and biocompatibility [8, 23]. There are three main types of vehicle: water-soluble substances, viscous, and oil-based [8]. In the present study, the vehicles of the different medicaments did not interfere in their tissue reaction since the results were similar. In the periods of 7 and 30 days, the number of giant cells was significantly reduced in the Calen group in comparison to UltraCal XS and Hydropast. At 30 days, the capsule adjacent to Hydropast showed a significantly lower number of giant cells than the UltraCal XS. The giant cells are derived from the fusion of 20 or more monocytes/macrophages and are formed for removing exogenous agents [24]. In the period of 30 days, the high number of giant cells verified in the UltraCal XS group suggests that this material may release more irritant substances than the Hydropast and Calen. 

In Calen paste, the calcium hydroxide is mixed to a viscous vehicle, polyethylene glycol 400, one of the most commonly used vehicles in root canal medicaments with low toxicity, high solubility in aqueous solutions, low immunogenicity and antigenicity [25], and antibacterial activity [5]. This vehicle releases calcium and hydroxyl ions more slowly and for longer periods than water-soluble and oil-based materials [26]. Because the releasing of H^+^, the polyethylene glycol 400 neutralizes the OH^−^ released by calcium hydroxide and, thereby, reduces the superficial necrosis area [27].

The propylene glycol is used as vehicle in the Hydropast; the vehicle used in the Hydropast is classified as a viscous vehicle with high molecular weight and, as well as polyethylene glycol 400, prolong the action of calcium hydroxide in the root canal system [26]. Moreover, propylene glycol shows low toxicity and antimicrobial properties [28, 29]. It was demonstrated that the addition of propylene glycol may not interfere in the biocompatibility of MTA in rat subcutaneous tissue [30]. 

Our results also suggest that the different radiopacifying agents of the pastes did not interfere in the tissue reaction. The endodontic materials should present sufficient radiopacity to be distinguished from adjacent anatomical structures, such as bone and teeth [31]. Zinc oxide, barium sulfate, bismuth oxide, and other components with iodine and bromine are some examples of radiopacifiers [12, 14, 27, 32]. 

The zinc oxide of Calen paste does not affect the biological properties of calcium hydroxide [27, 33]. Barium sulphate, radiopacifying agent of Hydropast, is also biocompatible because its cause no detrimental effect in rat subcutaneous tissue [34] or in periapical tissue in association with calcium hydroxide [14]; barium oxide in association with the Norian, an skeletal repair system (SRS), in tibiae defects of rats, maintains the properties of biocompatibility and osteoconductive materials of the SRS [35]. Although the manufacturer does not inform the radiopacifying agent of the UltraCal XS, our results demonstrated that this material has a good biological behavior. 

Considering the methodology used in the present study, our findings indicate that UltraCal XS and Hydropast are biocompatible in subcutaneous tissue of rats. 

## Figures and Tables

**Table 1:** Tested materials and composition.

	Composition	Manufacturer
Calen	49.77% calcium hydroxide, 50.23% of zinc oxide, colophony, and polyethylene glycol 400	S. S. White Artigos Dentários Ltda., Rio de Janeiro, RJ, Brazil

UltraCal XS	35% calcium hydroxide and a radiopacifier	Ultradent Products, Inc., South Jordan, UT, USA

Hydropast	38% of calcium hydroxide, 62% of barium oxide, and propilenoglicol	Biodinamica Quimica e Farmaceutica Ltda., Ibipora, PR, Brazil

**Table 2:** Number of inflammatory cells and giant cells/mm^2^ of the capsule adjacent to the implants in the subcutaneous.

*ߙ*	Inflammatory cells		Giant cells	
	7 days	30 days	7 days	30 days
Calen	2,795.10 ± 939.90^a,1^	960.64 ± 433.22^a,2^	4.14 ± 0.90^a,1^	0.92 ± 0.18^a,2^
UltraCal XS	2,397.66 ± 613.14^a,1^	975.99 ± 227.71^a,2^	6.00 ± 1.03^b,1^	2.77 ± 1.05^b,2^
Hydropast	2,248.10 ± 315.41^a,1^	1,407.82 ± 870.89^a,2 ^	8.77 ± 1.46^c,1^	1.37 ± 0.87^c,2^

Values are expressed as mean ± standard deviation.

Equal letters indicate no statistically significant difference (*P* > 0.05) between the materials in the same experimental period.

Different numbers indicate difference statistically significant (*P* ≤ 0.05) of each material in the different experimental periods.

## References

[1] Tronstad L (1992). Recent development in endodontic research. *Scandinavian Journal of Dental Research*.

[2] Siqueira JF (2001). Aetiology of root canal treatment failure: why well-treated teeth can fail. *International Endodontic Journal*.

[3] Faria G, Nelson-Filho P, Freitas AC, Assed S, Ito IY (2005). Antibacterial effect of root canal preparation and calcium hydroxide paste (Calen) intracanal dressing in primary teeth with apical periodontitis. *Journal of Applied Oral Science*.

[4] Leonardo MR, Hernandez MEFT, Silva LAB, Tanomaru-Filho M (2006). Effect of a calcium hydroxide-based root canal dressing on periapical repair in dogs: a histological study. *Oral Surgery, Oral Medicine, Oral Pathology, Oral Radiology and Endodontology*.

[5] Lima RKP, Guerreiro-Tanomaru JM, Faria-Júnior NB, Tanomaru-Filho M (2012). Effectiveness of calcium hydroxide-based intracanal medicaments against Enterococcus faecalis. *International Endodontic Journal*.

[6] Siqueira JF, Magalhães KM, Rôças IN (2007). Bacterial reduction in infected root canals treated with 2.5% NaOCl as an irrigant and calcium hydroxide/camphorated paramonochlorophenol paste as an intracanal dressing. *Journal of Endodontics*.

[7] Carrotte P (2004). Endodontics: part 9 Calcium hydroxide, root resorption, endo-perio lesions. *British Dental Journal*.

[8] Mohammadi Z, Dummer PMH (2011). Properties and applications of calcium hydroxide in endodontics and dental traumatology. *International Endodontic Journal*.

[9] Hasselgren G, Olsson B, Cvek M (1988). Effects of calcium hydroxide and sodium hypochlorite on the dissolution of necrotic porcine muscle tissue. *Journal of Endodontics*.

[10] Safavi KE, Nichols FC (1993). Effect of calcium hydroxide on bacterial lipopolysaccharide. *Journal of Endodontics*.

[11] Tanomaru JMG, Leonardo MR, Tanomaru Filho M, Bonetti Filho I, Silva LAB (2003). Effect of different irrigation solutions and calcium hydroxide on bacterial LPS. *International Endodontic Journal*.

[12] Alaçam T, Görgül G, Ömürlü H (1990). Evaluation of diagnostic radiopaque contrast materials used with calcium hydroxide. *Journal of Endodontics*.

[13] Fava LRG, Saunders WP (1999). Calcium hydroxide pastes: classification and clinical indications. *International Endodontic Journal*.

[14] Orucoglu H, Cobankara FK (2008). Effect of unintentionally extruded calcium hydroxide paste including barium sulfate as a radiopaquing agent in treatment of teeth with periapical lesions: report of a case. *Journal of Endodontics*.

[15] Heward S, Sedgley CM (2011). Effects of intracanal mineral trioxide aggregate and calcium hydroxide during four weeks on ph changes in simulated root surface resorption defects: an in vitro study using matched pairs of human teeth. *Journal of Endodontics*.

[16] Blanscet ML, Tordik PA, Goodell GG (2008). An agar diffusion comparison of the antimicrobial effect of calcium hydroxide at five different concentrations with three different vehicles. *Journal of Endodontics*.

[17] International Organization for Standardization

[18] Pereira MSS, Faria G, da Silva LAB, Tanomaru-Filho M, Kuga MC, Rossi MA (2012). Response of mice connective tissue to intracanal dressings containing chlorhexidine. *Microscopy Research and Technique*.

[19] Viola NN, Guerreiro-Tanomaru JM, da Silva GF, Sasso-Cerri E, Tanomaru-Filho M, Cerri PS (2012). Biocompatibility of an experimental MTA sealer implanted in the rat subcutaneous: quantitative and immunohistochemical evaluation. *Journal of Biomedical Materials Research B*.

[20] Faria G, Celes MRN, de Rossi A, Silva LAB, Silva JS, Rossi MA (2007). Evaluation of chlorhexidine toxicity injected in the paw of mice and added to cultured L929 fibroblasts. *Journal of Endodontics*.

[21] Guerreiro-Tanomaru JM, Chula DG, de Pontes Lima RK, Berbert FLVC, Tanomaru-Filho M (2012). Release and diffusion of hydroxyl ion from calcium hydroxide-based medicaments. *Dental Traumatology*.

[22] Holland R, Pinheiro CE, de Mello W, Nery MJ, de Souza V (1982). Histochemical analysis of the dogs’ dental pulp after pulp capping with calcium, barium, and strontium hydroxides. *Journal of Endodontics*.

[23] Ballal NV, Shavi GV, Kumar R, Kundabala M, Bhat KS (2010). In vitro sustained release of calcium ions and pH maintenance from different vehicles containing calcium hydroxide. *Journal of Endodontics*.

[24] Cerri PS, Freymüller E, Katchburian E (1997). Light and electron microscopic study of autologous implants of dental roots in the subcutaneous tissue of rats. *The Bulletin of Tokyo Dental College*.

[25] Athanassiadis B, Abbott PV, Walsh LJ (2007). The use of calcium hydroxide, antibiotics and biocides as antimicrobial medicaments in endodontics. *Australian Dental Journal*.

[26] Gomes BP, Ferraz CC, Vianna ME (2002). In vitro antimicrobial activity of calcium hydroxide pastes and their vehicles against selected microorganisms. *Brazilian dental journal*.

[27] Nelson Filho P, Silva LA, Leonardo MR, Utrilla LS, Figueiredo F (1999). Connective tissue responses to calcium hydroxide-based root canal medicaments. *International Endodontic Journal*.

[28] Bhat KS, Walkevar S (1975). Evaluation of bactericidal property of propylene glycol for its possible use in endodontics. *Arogya Journal of Health Science*.

[29] Thomas PA, Bhat KS, Kotian KM, Manipal SK (1980). Antibacterial properties of dilute formocresol and eugenol and propylene glycol. *Oral Surgery Oral Medicine and Oral Pathology*.

[30] Holland R, Mazuqueli L, de Souza V, Murata SS, Dezan Júnior E, Suzuki P (2007). Influence of the type of vehicle and limit of obturation on apical and periapical tissue response in dogs’ teeth after root canal filling with mineral trioxide aggregate. *Journal of Endodontics*.

[31] Pires FCS, Pardini LC, Cruvinel DR, Hamida HM, Garcia LF (2012). In vitro comparison of the radiopacity of cavity lining materials with human dental structures. *Journal of Conservative Dentistry*.

[32] Cornélio ALG, Salles LP, Campos da Paz M, Cirelli JA, Guerreiro-Tanomaru JM, Tanomaru Filho M (2011). Cytotoxicity of Portland cement with different radiopacifying agents: a cell death study. *Journal of Endodontics*.

[33] de Queiroz AM, Assed S, Consolaro A (2011). Subcutaneous connective tissue response to primary root canal filling materials. *Brazilian Dental Journal*.

[34] Adams DO (1976). The granulomatous inflammatory response: a review. *American Journal of Pathology*.

[35] Acarturk O, Lehmicke M, Aberman H, Toms D, Hollinger JO, Fulmer M (2008). Bone healing response to an injectable calcium phosphate cement with enhanced radiopacity. *Journal of Biomedical Materials Research B*.

